# Exploring New Sources of Bioactive Phenolic Compounds from Western Balkan Mountains

**DOI:** 10.3390/plants11071002

**Published:** 2022-04-06

**Authors:** Erna Karalija, Sabina Dahija, Arnela Demir, Renata Bešta-Gajević, Sanja Ćavar Zeljković, Petr Tarkowski

**Affiliations:** 1 Laboratory for Plant Physiology, Faculty of Science, University of Sarajevo, Zmaja od Bosne 33-35, 71 000 Sarajevo, Bosnia and Herzegovina; erna.k@pmf.unsa.ba (E.K.); sabina.dahija@pmf.unsa.ba (S.D.); arnela.demir@pmf.unsa.ba (A.D.); 2 Laboratory for Microbiology, Faculty of Science, University of Sarajevo, Zmaja od Bosne 33-35, 71 000 Sarajevo, Bosnia and Herzegovina; renata.bg@pmf.unsa.ba; 3 Centre of Region Haná for Biotechnological and Agricultural Research, Czech Advanced Technology and Research Institute, Palacky University, Šlechtitelů 27, 78371 Olomouc, Czech Republic; petr.tarkowski@upol.cz; 4 Centre of the Region Haná for Biotechnological and Agricultural Research, Department of Genetic Resources for Vegetables, Medicinal and Special Plants, Crop Research Institute, Šlechtitelů 29, 78371 Olomouc, Czech Republic

**Keywords:** *Valeriana montana* L., *Salix retusa* L., *Campanula hercegovina* Degen and Fiala, phenolic profile, antioxidant activity, antimicrobial activity

## Abstract

This study presents the first report on phenolic composition and bioactivity of ethanolic extracts of three plant species that grow in the western Balkan mountains and are used in traditional folk medicine: *Valeriana montana*, *Salix retusa*, and *Campanula hercegovina*. Phenolics were extracted from different aerial plant parts using 80% ethanol to assess the possibility of sustainable use of these plants as a source of bioactive compounds without disruption to the roots (for *V. montana*) or destruction of whole habitats (for *S. retusa* and *C. hercegovina*). The ethanolic extract of *V. montana* flower contained noticeable levels of apigenin and quercetin. The branches and bark of *S. retusa* were significantly rich in catechin, while rutin was the major phenolic found in the leaf extract of *C. hercegovina*. Furthermore, the flower extract of *V. montana* revealed the best antioxidant activity, which was comparable to 4-hydroxybenzoic acid and quercetin. Considering antimicrobial activity, the leaf extracts of *V. montana* and *C. hercegovina* demonstrated potent activity against all microbes tested, while the extracts of *S. retusa* were moderately effective. The presented results emphasize the potential of these plants as novel sources of bioactive compounds.

## 1. Introduction

Plants are rich sources of a wide variety of bioactive phenolic compounds, which have many beneficial effects for humans and animals. Many plant species contain significant amounts of phenolics and have been used for centuries in treatments of different disorders. The interest in phenolic compounds has increased during the last few decades due to their antioxidant potential [1], which can help in the prevention of chronic and oxidative stress-related diseases, such as cancer, cardiovascular, and neurodegenerative disorders [2]. In a world where antibiotic resistance is real, the search for new antimicrobials often leads to plant sources [3], because plant extracts that contain many different compounds act more efficiently than a single compound. This effect is called the synergistic effect. Natural compounds from plant extracts can synergize by targeting multiple receptors, facilitating transport to a target, and offering protection from degradation as well as modification of resistance [4]. Although it is difficult to prove the activity of the compounds in complex mixtures, synergistic interactions in plant extracts are evidenced by the frequent loss of activity upon fractionation [5,6,7]. However, only 20% of known plants have been investigated in pharmaceutical studies [8], and still many species must be inspected. Therefore, in a continuation of our research on medicinal and aromatic plants of the Balkan Peninsula [9,10,11], in this paper, we present the phytochemical profiles and some biological activities of *Valeriana montana* L., *Salix retusa* L., and *Campanula hercegovina* Degen and Fiala. The phytochemistry of these species was not investigated until today.

The genus *Valeriana* includes species commonly named valerians, with a long history of medicinal use as sedatives. Roots and rhizomes of many valerians (*V. officinalis* L., *V. wallichii* DC., *V. edulis* Nutt., etc.) are used as phytotherapeutics with the effect of a mild sedative, and as treatments for insomnia, seizures, anxiety, etc. [12,13,14]. Many valerian species face excessive exploitation and a decrease in natural populations [15], and recent studies confirming broader bioactive properties of valerians, such as antimicrobial and antioxidant activity [16,17,18], contribute to the overexploitation of valerian roots. *Valeriana montana*, the dwarf valerian, a perennial plant with a herbaceous stem bearing bell- or funnel-shaped, whitish to pink flowers, is native to Europe. The plant can be found in mountains from 800 to 1600 m above sea level [19]. The roots of this plant are collected traditionally in the western Balkan region for their medicinal properties, while above-ground parts are discarded. This species is poorly investigated, with no record of secondary metabolite composition or biological activity report; thus, no confirmed bioactive properties can be attributed to this species, making it interesting for our study. 

The genus *Salix* L. (willows) belongs to the Salicaceae family and includes 330–500 species of trees and shrubs with simple, stipulate leaves alternately arranged on woody stems [20]. The medicinal and economic value of willows are exploited in traditional medicine as well as in pharmacy, and willow-originated metabolites are known to be used in the food industry as natural additives and as ingredients in cosmetic products, including sunscreen and anti-aging cream formulations [21,22,23]. Willows are mostly known for their high levels of salicylates, becoming a major object of research after the discovery of aspirin. The pharmacological value of willows does not only arise from the salicin portion in the willow bark but from other components as well such as polyphenolics. It is considered that antioxidant active ingredients such as polyphenols in willow bark contribute to the anti-inflammatory activity [24,25]. *Salix retusa* is a small species (height up to 30 cm) mainly found in the mountains of central and southern Europe. The study presented here investigates the potential of *Salix retusa* as a new natural source of phenolic compounds with bioactive potential. Although this species belongs to the popular willow genus, its chemical composition remains unknown.

The genus *Campanula* includes more than 400 species mainly distributed in the northern hemisphere. The majority of the species are endemic to the Mediterranean areas, meadows, and mountain hills. All species are herbaceous, and their name, bellflowers, refers to the bell-shaped and, in most species, blue flowers [26]. Although members of this genus are mainly cultivated for ornamental purposes, several members, including *Campanula glomerata*, *C. persicifolia*, *C. rotundifolia*, *C. bononiensis*, *C. sibirica*, and *C. patula* have been used locally for the preparation of traditional drugs in Russian and Italian folk medicine [27]. However, phytochemical investigations of this genus only include a few reports on flavonoids and anthocyanins from flowers [28,29], as well as their very potent antioxidant activity [30,31]. *Campanula hercegovina* is an endemic species [32] in the region from the Dinaric Alps to Albania. It grows on limestone rocks in mountainous areas. As mentioned above, the chemistry of this species remains unknown.

Sustainable harvesting and cultivation of wild-growing species are two measures that can be employed in the preservation of species germplasm [33] in areas where wild plant gathering is embedded in traditional medicine such as the western Balkans. The presented research aims to evaluate the bioactive potential of the above-ground parts of *V. montana*, *S. retusa*, and *C. hercegovina* for better and more complete use and sustainable exploitation of these species. 

## 2. Results

In this study, the phenolic composition of three investigated species is presented for the first time (Table 1).

Results show that *V. montana* contained the least amounts of phenolic compounds among all three plants investigated, while *S. retusa* was significantly rich in these phytochemicals. Additionally, the flower extract of *V. montana* contained noticeable levels of apigenin (32.50 ± 0.94 mg/g) and quercetin (43.76 ± 2.12 mg/g), while these flavonoids were not detected in the extracts of other plants. The branches and bark of *S. retusa* were significantly rich in catechin, a natural antioxidant with a wide range of biological activities [34]. The concentrations of this flavonoid ranged from 359.97 ± 15.97 mg/g in the leaf extract to 843.62 ± 17.44 mg/g in the extract of old branches. The young branches and leaves of *S. retusa* were also rich in chlorogenic acid, with concentrations of 453.00 ± 25.36 mg/g and 438.97 ± 79.64 mg/g, respectively. Rutin was the major phenolic found in the leaf extract of *C. hercegovina* (205.33 ± 25.50 mg/g). Both flower and leaf extracts of this endangered species contained noticeable levels of chlorogenic acid i.e., 32.70 ± 0.66 and 55.55 ± 2.00 mg/g, respectively. The stems of this species did not contain significant levels of any phenolic compound detected (Table 1). In general, extracts of *S. retusa* contain the most phenolic compounds, while the extracts of *V. montana* contain the least. Specifically, identified phenolics from *S. retusa* extracts reached 58.96% to 86.37% of the total, while the extracts of aerial parts of *V. montana* reached only up to 8.61%.

Furthermore, the biological properties of these extracts were assayed in terms of antioxidant and antimicrobial activities (Table 2). According to the presented results, the flower extract of *V. montana* revealed the lowest IC_50_ value for antioxidant activity (48.13 ± 0.86 μg/mL), which is comparable to 4-hydroxybenzoic acid (IC_50_ 45.60 ± 0.41 μg/mL) and quercetin (IC_50_ 38.49 ± 1.90 μg/mL), which were also assayed. Other examined extracts showed relatively similar antioxidant potential, with IC_50_ values of 89.65 ± 0.61 and 129.21 ± 1.70 μg/mL for the leaf and flower extracts of *C. hercegovina*, respectively, while the stem extract of the same species revealed the lowest ability to scavenge free radicals (Table 2).

The examined extracts and phenolic compounds did not have significant activity against *Salmonela aboni*. However, the leaf extract of *V. montana* revealed the best activity, which was better than assayed standards of phenolic compounds. In general, the leaf extract of *V. montana,* as well as the leaf extract of *C. hercegovina*, showed the best activity against all microbes used, while all extracts of *S. retusa* were moderately effective. Regarding analyzed phenolic compounds found in these plant extracts, salicylic, chlorogenic, and ferulic acids revealed very potent antioxidant activities against stable radicals, reaching IC_50_ values of 2.71 ± 0.04, 5.62 ± 0.02, and 7.36 ± 0.10 μg/mL, respectively. The flavonoid quercetin showed weaker antioxidant properties, but was very potent against *Enterococcus faecalis*, *Staphylococcus aureus*, and also *Candida albicans* (Table 2). Considering the effectiveness of the extract compared to the antibiotic or antimycotic, *Valeriana montana* leaf extracts showed great efficiency against *Esherichia coli*, and flower extracts against *Candida albicans*.

**Figure 1:**
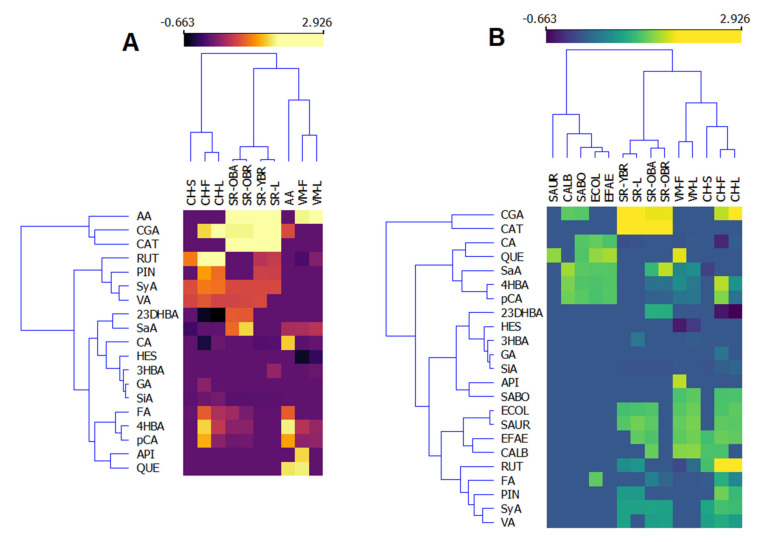
Heatmaps of the phenolic composition and antioxidant (**A**) and antimicrobial (**B**) activities of investigated extracts of *Valeriana montana* (VM), *Salix retusa* (SR), and *Campanula hercegovina* (CH). S—stems, F—flowers, L—leaves, OBA—old branches bark, OBR—old branches, YBR—young branches, AA—antioxidant activity, SAUR—*S. aureus*, CALC—*C. albicans*, ECOL—*E. coli*, EFAE—*E. faecalis*, CGA—chlorogenic acid, CAT—catechin, RUT—rutin, PIN—pinocembrin, SyA—syringic acid, VA—vanillic acid, 23DHBA—2,3-dihydroxybenzoic acid, SaA—salicylic acid, CA—caffeic acid, HES—hesperidin, 3HBA—3-hydroxybenzoic acid, GA—gallic acid, SiA—sinapic acid, FA—ferulic acid, 4HBA—4-hydroxybenzoic acid, pCA—*p*-coumaric acid, API—apigenin, QUE—quercetin.

As mentioned above, the bioactivity of plant extracts, as complex mixtures of different classes of natural compounds, is often better than the bioactivity of a single isolated bioactive compound [4,5,6,7]. However, it is important to know which compounds are the carriers of these activities. Therefore, we constructed heatmaps of the correlation between the composition of the investigated extracts and their antioxidant (Figure 1A) and antimicrobial activities (Figure 1B). Logarithmic values of the data presented in Table 1 and Table 2 were used to construct these heatmaps. According to the heatmaps, the phenolic composition of investigated plants is significantly different, and the plant extracts are separated into clusters (Figure 1). Considering the antioxidant activity (AA), it is clear that both catechin (CAT) and chlorogenic acid (CGA) are the main radical scavengers in the investigated plant extracts, as they are grouped in the same cluster as the antioxidant activity of the extracts (Figure 1A). Furthermore, as presented in Table 2, extracts of *V. montana* (VM) show the best antioxidant activity and are also clustered together, but apigenin and quercetin do not appear to contribute significantly to the radical-scavenging activity of these extracts. The second group contains the extracts of *S. retusa*, which also show prominent antioxidant activity. The extracts of *C. hercegovina* are separated into another cluster with a longer Euclidian distance from the antioxidant activity, meaning they are weaker antioxidants. However, the carriers of the antioxidant activities oin the extracts of *C. hercegovina* seem to be rutin and pinocembrin, as well as syringic and vanillic acids. Considering antimicrobial activities, extracts of *S. retusa* show the best activities among the others (Figure 1B). The most used microbes are clustered together with rutin and pinocembrin, and with ferulic, synaptic, and vanillic acids, so it can be concluded that these phenolic compounds are the main carriers of the antimicrobial activities of the extracts.

## 3. Discussion

This study presents, for the first time, the phytochemical composition of aerial parts of three species from the western Balkan mountains, i.e., *Valeriana montana*, *Salix retusa*, and *Campanula hercegovina*. Eighteen phenolic compounds, including hydroxyphenolic acids (2,3-dihydoxybenzoic, gallic, 3-hydroxybenzoic, 4-hydroxybenzoic, salicylic, syringic, and vanillic acid), hydroxycinnamic acids (caffeic, chlorogenic, *p*-coumaric, ferulic, and sinapic acid), and flavonoids (apigenin, catechin, hesperetin, pinocembrin, quercetin, and rutin) were identified and quantified in the investigated ethanolic extracts of these plants. Significant differences between plant organs were noticed considering qualitative and quantitative composition (Table 1).

There are very few investigations into the bioactive potential of valerians beyond the activities associated with their roots. The presented research provides valuable insight into the value of valerians beyond their roots and allows exploration of the sustainable exploitation of these plants by preserving the root in natural habitats and using the aerial plant parts as bioactive compound sources. Extracts from *V. montana* were diverse and their compositions were correlated to the plant part used for extraction (Table 1). A previous study on *V. jatamansi* leaf extracts showed that the highest content of phenolics is found at the pre-flowering and flowering stages [18]. The levels of phenolic acids of *V. jatamansi* were similar to those reported here for *V. montana*. However, these authors did not report the concentrations of any flavonoid in *V. jatamansi*, while *V. montana* contains significant amounts of apigenin and quercetin. In addition, methanolic extracts of *V. dioscoridis* were found to be rich in chlorogenic acid and hesperidin [34], which is not the case for *V. montana* (Table 1). The significant content of flavonoids in aerial plant parts of *V. montana* provides evidence for the biological potential of the whole plant, suggesting the possibility of extracting bioactive compounds from plant parts that are usually discarded during root extraction preparations. Flower extracts of *V. montana* showed a very potent ability to scavenge free radicals (Table 2), which is in agreement with the available data on other *Valeriana* species [16,18,34]. Only a few works are available regarding the possible antimicrobial activity of valerians. One is a study on the antibacterial potential of *V. jatamansi* shoot extract for Ag-metal bioreduction and its antimicrobial activity [35]. Ag-nano particles *V. jatamansi* were tested against bacteria and fungi in combination with recorded antimicrobial activity [18]. In the case of *V. wallichii*, strong antimicrobial properties were recorded for chloroform fraction against *Bacillus subtilus* and *S. aureus* [36]. Considering the effectiveness of *V. montana*, it is comparable to antibiotics and antimycotics, with a lower chance of bacteria becoming resistant to plant extracts in comparison to emerging antibiotic resistance. Due to the complexity of the chemical composition of plant extracts, diverse chemical structures, and mechanisms of action of these compounds, the antimicrobial effect of phytochemicals relies on a complex interaction of different compounds [37]. For this reason, phytochemicals and plant extracts can be effective against multiple drug-resistant bacteria, as described in previous studies [38,39].

The amount of catechin was significant in all investigated extracts of *S. retusa*. Catechins are natural antioxidants, with a wide range of biological activities, such as antifungal, antiviral, anti-inflammatory, cancer prevention, and many others [40]. They are commonly found in different plants (green tea, cocoa beans, fruits, etc.) and have been previously recorded in willows with content ranging from 0.108 (*S. amygdalina*) to 1.97 nM/mg of extract (*S. fragilis*), which is much lower compared to the content recorded here in *S. retusa* [41]. The previously published antibacterial effect of *Salix babylonica* bark extract against *Escherichia coli* and *Salmonella enterica* recorded similar efficiency compared to the used antibiotic [42]. Bark extracts of *Salix alba* were moderately potent against *Staphylococcus aureus*, with low activity against *Escherichia coli* [43]. The antimicrobial potential of *Salix alba* is correlated to tannin content, with the most effective tannins against *Staphylococcus aureus* found in bark extracts [44]. There is no data regarding any bioactive properties of *S. retusa*; this study represents the first investigation of the antioxidant and antibacterial potential of this species. Extracts of this plant had a moderate effect on all tested microbes, except where no activity was recorded with *S. aboni*. Leaf extract was highly efficient against *S. aureus*, while bark extract was efficient against *C. albicans*. The amount of chlorogenic acid in leaf extract was presumably responsible for its effect against *S. aureus*, because chlorogenic acid can inhibit the sortase A enzyme in this bacterium [45]. In contrast, high amounts of catechin in the bark were presumably responsible for its effect against *C. albicans*, as recorded by previous studies [46,47]. Interestingly, a similar composition was recorded for extracts from old branches, but no antimicrobial activity was recorded. The presence of ferulic acid in bark extracts could have influenced the antimicrobial efficiency through a synergistic effect when combined with catechin [48].

Aerial parts of *C. hercegovina* contained significant amounts of rutin and pinocembrin, as well as chlorogenic acid (Table 1), which are also found in other species of *Campanula* genus [49,50]. However, only 30% of phenolic compounds were identified in the leaf extract of this bellflower (Table 1), but the data in the literature indicate that the major compounds in *Campanula* leaves are luteolin derivatives, which may be used as chemotaxonomic markers [51]. The antioxidant activity of other *Campanula* species has been described in the literature [49,50], and was generally lower than presented here. The IC_50_ value of DPPH radical-scavenging activity of the methanolic extract of *C. latifolia* susp. *latifolia* was 410.67 ± 2.49 μg/mL [49], while the methanolic extract of *C. macrostachya* reached an IC_50_ value of 4150 μg/mL [50]. In addition, Alhage et al. [52] showed that the leaf and flower extracts of *C. retrorsa* were active against *C. albicans*, while the extracts of *C. glomerata* and *C. olympica* were potent against *K. pneumonias* [53] but were inefficient against *E. coli* and *S. aureus*.

## 4. Materials and Methods

### 4.1. Plant Material

The aerial parts of *Salix retusa* L. and *Valeriana montana* L. were collected in the summer of 2020 on Mt Bjelašnica (close to Sarajevo, Bosnia and Herzegovina; 43.7042° N, 18.2567° E), while samples of *Campanula hercegovina* (A. Degen) were collected at Mt Rujište (close to Mostar, Bosnia and Herzegovina; 43.4507° N, 17.9745° E). The voucher specimens were deposited at the Herbarium collection of the Department of Biology, Faculty of Science, the University of Sarajevo, labelled *S. retusa* LRPER 371, *V. montana* LFB377, and *C. hercegovina* LFB372. The material of *S. retusa* was separated into the bark of the old branches, the wood of the old branches, whole young branches (one-year old), and leaves. Inflorescences and leaves of *V. montana* were also separated, while aerial parts of *C. hercegovina* were separated into flowers, leaves, and stems.

The material was air-dried to constant mass at room temperature while protected from light and subsequently stored at +4 °C until use.

### 4.2. Preparation of Plant Extracts and Reference Solutions

Dried plant samples were ground into a powder in a TissueLyser (Qiagen, Hilden, Germany); subsequently, 2 grams of the powder was mixed with 10 mL of 80% ethanol and sonicated for 30 min at 23 °C, followed by centrifuge at 5000 rpm 15 min. The supernatant was collected, and the remaining pellet was re-extracted using 10 mL 80% ethanol. Both supernatants were combined for further analysis. 

### 4.3. UHPLC–MS/MS Analysis

Plant extracts were analyzed for polyphenolic composition via LC–MS/MS according to the previously published method [54]. Briefly, LC–MS/MS measurements were carried out using an Ultra Performance LCMS 8050 system (Shimadzu, Kyoto, Japan) with a triple quadrupole mass spectrometer equipped with an electrospray ionization (ESI) source operating in the negative mode. The samples were injected into a reversed-phase column (Acquity UPLC BEH C18, 1.7 μm, 2.1 × 150 mm, Waters, Milford, MA, USA) with an appropriate pre-column (Acquity UPLC BEH C18 VanGuard Pre-column, 1.7 µm, 2.1 mm × 5 mm). The mobile phase consisted of a mixture of aqueous solutions of 0.1% formic acid in water (solvent A) and 0.1% formic acid in methanol (solvent B) at a flow rate of 0.4 mL/min. The linear gradient consisted of 5% B for 3 min, 5–25% B for 4 min, 25–30% B for 6 min, 30–35% B for 4 min, 35–60% B for 6 min, 60–100% for 4 min, isocratic for 1.5 min, back to 5% B within 0.1 min, and equilibration for 3.4 min.

The effluent was introduced into an electrospray source (interface temperature of 300 °C, heat block temperature of 400 °C, and capillary voltage of 3.0 kV). To achieve high specificity in addition to the high sensitivity, the analysis was performed in the multiple reaction monitoring (MRM) mode. All standards and reagents were of the highest available purity and purchased from Sigma Aldrich Company (Prague, Czech Republic), and the measurements were performed in triplicate.

### 4.4. Antioxidant Activity

DPPH (2.2-diphenyl-1-picrylhydrazyl radical) antioxidant activity was evaluated for all extracts according to Meda et al. [55]. First, 100 μL of the sample extract was mixed with 900 μL of DPPH 96% ethanol and incubated in the dark for 30 min, followed by an absorbance reading at 517 nm against blank. A solution of 96% ethanol was used to zero the spectrophotometer, DPPH solution was used as a blank sample, and different phenolic compounds found in the investigated extracts (Table 1 and Table 2) were used as positive probes.

Antioxidant potential was evaluated according to the absorbance change against the blank, which contained ethanol instead of an extract, at different dilution levels and presented as IC_50_ values (concentration of extract necessary for 50% DPPH radical inhibition).

### 4.5. Antimicrobial Properties

The agar-well diffusion method was used to evaluate the antimicrobial activity of plant extracts and standards according to the National Committee for Clinical Laboratory Standards [56]. Each well contained 100 μL of extract (0.1 mg/mL). Bacterial strains used in the analysis included Gram-positive bacteria, i.e., *Enterococcus faecalis* ATCC^®^ 19433^TM^, *Staphylococcus aureus* subsp. *aureus* ATCC^®^ 6538^TM^; Gram-negative bacteria, i.e., *Salmonella abony* NCTC^®^ 6017^TM^, *Escherichia coli* ATCC^®^ 8739^TM^; and the yeast *Candida albicans* ATCC^®^ 10231^TM^. Bacterial strains were used as standardized inoculum of 5 × 10^5^ CFU/mL using the McFarland standard. Müller–Hinton broth and Sabouard medium were used to cultivate bacterial strains and yeast, respectively. Both plant extracts and selected phenolic compounds detected in them (Table 1 and Table 2) were assayed for their antimicrobial properties. Ampicillin was used as a positive standard for bacterial strains and nystatin for the yeast. A solution of 80% ethanol was used as a negative control. Antimicrobial effect was expressed as a diameter of inhibition zone in mm reduced by values given by negative control.

### 4.6. Statistical Analysis

All data were analyzed using the STATISTICA 10.0 software (Statsoft Inc., Tulsa, OK, USA) and in Orange data mining software v.3.31.1. Experimental results were presented in tables and graphs as the mean ± standard deviation of three independent replications. Obtained data were subjected to variance analysis (ANOVA) and the Newman–Keuls post hoc test was performed to identify significant differences between extract types. Mean values with *p* < 0.01 were considered statistically significant. Pearson correlations were performed to observe the possible correlation between the phenolic profile, antioxidant capacity, and detected antimicrobial activity at the level of significance *p* < 0.01.

## 5. Conclusions

In the presented research, we evaluated different plant parts of three selected plant species (*Valeriana montana*, *Salix retusa*, and *Campanula hercegovina*) that grow wild in the western Balkans for their bioactive properties and concluded that all three species are rich in bioactive compounds. The most interesting was the bioactive potential of aerial parts of *V. montana*, which were rich in flavonoids apigenin and quercetin. In addition, extracts of *S. retusa* were rich in catechin and showed moderate bioactivity, while extracts of *C. hercegovina* contained significant levels of rutin with potent activity against all tested microbes. Presented results indicate the possibility of the use of these species without disrupting the roots, resulting in more sustainable exploitation. 

## Figures and Tables

**Table 1:** Phenolic profiles of investigated plant extracts (mg/g).

Compound	VM-F	VM-L	SR-B	SR-OB	SR-YB	SR-L	CH-F	CH-L	CH-S
2,3-Dihydoxybenzoic acid	nd	nd	nd	nd	6.94 ^d^ ± 1.60	nd	0.30 ^e^ ± 0.00	0.22 ^f^ ± 0.08	nd
Gallic acid	nd	nd	nd	nd	nd	nd	1.80 ^e^ ± 0.02	nd	nd
3-Hydroxybenzoic acid	nd	1.11 ^c^ ± 0.09	nd	nd	nd	1.95 ^c^ ± 0.39	nd	nd	nd
4-Hydroxybenzoic acid	3.22 ^c^ ± 0.03	2.06 ^b^ ± 0.52	nd	nd	1.77 ^e^ ± 0.10	nd	31.24 ^b^ ± 4.97	3.75 ^d,e^ ± 0.17	nd
Salicylic acid	2.79 ^c^ ± 0.01	3.31 ^b^ ± 0.89	nd	8.79 ^b^ ± 0.05	32.33 ^c^ ± 0.95	nd	nd	nd	0.65 ^c^ ± 0.00
Syringic acid	nd	nd	5.33 ^b^ ± 0.13	5.18 ^c^ ± 0.04	5.26 ^d^ ± 0.05	5.19 ^b^ ± 0.03	10.85 ^d^ ± 1.51	9.75 ^c^ ± 0.30	5.70 ^b^ ± 0.37
Vanillic acid	nd	nd	4.83 ^b^ ± 0.35	5.14 ^c^ ± 0.54	5.31 ^d^ ± 0.73	nd	6.65 ^d^ ± 1.23	4.83 ^d^ ± 0.81	4.97 ^b^ ± 0.89
Caffeic acid	0.93 ^e^ ± 0.12	1.05 ^c,d^ ± 0.15	nd	nd	nd	0.88 ^d^ ± 0.04	0.39 ^e^ ± 0.01	1.15 ^f^ ± 0.05	nd
Chlorogenic acid	nd	nd	nd	nd	48.39 ^b^ ± 0.95	438.97 ^a^ ± 79.64	32.70 ^b^ ± 0.66	55.55 ^b^ ± 2.00	nd
*p*-Coumaric acid	1.84 ^d^ ± 0.12	1.93 ^b,c^ ± 0.02	nd	nd	1.25 ^e^ ± 0.03	nd	20.20 ^c^ ± 3.73	1.82 ^e,f^ ± 0.07	nd
Ferulic acid	nd	nd	2.35 ^c^ ± 0.08	nd	1.40 ^e^ ± 0.03	nd	7.11 ^d^ ± 1.16	2.85 ^e^ ± 0.02	1.06 ^c^ ± 0.03
Sinapic acid	nd	nd	0.91 ^d^ ± 0.01	0.92 ^d^ ± 0.01	0.93 ^e^ ± 0.02	0.93 ^d^ ± 0.01	1.17 ^e^ ± 0.02	1.33 ^f^ ± 0.06	0.94 ^c^ ± 0.05
Apigenin	32.50 ^b^ ± 0.94	nd	nd	nd	nd	nd	nd	nd	nd
Catechin	nd	nd	838.39 ^a^ ± 210.13	843.62 ^a^ ± 157.44	485.97 ^a^ ± 6.86	359.97 ^a^ ± 45.97	nd	nd	nd
Hesperetin	0.32 ^e^ ± 0.01	0.54 ^d^ ± 0.15	nd	nd	nd	nd	nd	nd	nd
Pinocembrin	nd	nd	nd	nd	nd	4.36 ^b^ ± 0.31	16.72 ^c^ ± 0.93	9.18 ^c^ ± 0.86	nd
Quercetin	43.76 ^a^ ± 2.12	nd	nd	nd	nd	nd	nd	nd	nd
Rutin	0.75 ^e^ ± 0.01	1.54 ^d^ ± 0.52	nd	nd	nd	3.98 ^b^ ± 1.23	82.74 ^a^ ± 3.49	205.33 ^a^ ± 25.50	11.14 ^a^ ± 0.24
Total identified (%)	8.61	1.15	85.18	86.37	58.96	81.62	21.19	29.57	2.45

VM—*Valeriana montana*; SR—*Salix retusa*; CH—*Campanula hercegovina*; F—flowers; L—leaves; B—barks; OB—old branches; YB—young branches; S—stems; nd—not detected. The data represent the means of three replicates (±standard deviation). The values within one column followed by the same letter do not differ significantly after the ANOVA post hoc Newman–Keuls analysis at a significance level of *p* < 0.01.

**Table 2:** Bioactive properties of the investigated plant extracts.

Plant Extract/ Compound	Antioxidant Activity	Antimicrobial Activity (mm)				
	IC_50_ (μg/mL)	*S. aboni*	*E. coli*	*E. faecalis*	*S. aureus*	*C. albicans*
VM-F	48.13 ^e^ ± 0.86	11.67 ^c^ ± 0.58	13.33 ^c^ ± 0.58	14.33^d^ ± 1.15	15.00 ^c,d^ ± 1.00	20.33 ^b^ ± 0.58
VM-L	95.02 ^c^ ± 1.35	14.00 ^b^ ± 1.73	15.67 ^a^ ± 2.89	17.00 ^c^ ± 1.00	18.00 ^c^ ± 3.46	21.33 ^b^ ± 1.15
SR-B	94.41 ^c^ ± 0.26	nd	12.33 ^c,d^ ± 1.15	12.00 ^e^ ± 1.00	14.00 ^d^ ± 0.00	15.33 ^c,d^ ± 1.15
SR-OB	92.96 ^c,d^ ± 0.15	nd	nd	nd	nd	nd
SR-YB	93.83 ^c^ ± 0.25	nd	11.00 ^d^ ± 0.00	nd	13.00 ^d^ ± 1.73	nd
SR-L	94.22 ^c^ ± 0.05	nd	11.33 ^d^ ± 0.58	14.67 ^d^ ± 0.58	17.33 ^c^ ± 0.58	nd
CH-F	129.21 ^b^ ± 1.70	11.33 ^c^ ± 0.58	11.67 ^d^ ± 1.15	15.67 ^d^ ± 1.15	14.33 ^d^ ± 0.58	11.33 ^e^ ± 1.15
CH-L	89.65 ^d^ ± 0.61	11.67 ^c^ ± 1.15	14.33 ^a,c^ ± 1.15	14.67 ^d^ ± 1.15	13.67 ^d^ ± 0.58	nd
CH-S	223.25 ^a^ ± 4.75	nd	nd	10.67 ^f^ ± 0.58	nd	11.67 ^e^ ± 1.15
Chlorogenic acid	5.62 ^h^ ± 0.02	13.00 ^b,c^ ± 0.00	nd	nd	nd	14.33 ^d^ ± 0.58
Ferulic acid	7.36 ^h^ ± 0.10	nd	14.67 ^a,c^ ± 0.58	nd	nd	nd
*p*-Coumaric acid	17.76 ^g^ ± 1.09	13.67 ^b,c^ ± 0.58	11.33 ^d^ ± 0.58	13.00 ^e^ ± 0.00	nd	17.00 ^c^ ± 0.00
Salicylic acid	2.71 ^h^ ± 0.04	13.50 ^b,c^ ± 0.71	13.00 ^b,c^ ± 1.73	13.00 ^e^ ± 1.73	nd	25.33 ^a^ ± 0.58
4-Hydroxybenzoic acid	45.60 ^e^ ± 0.41	12.33 ^b,c^ ± 1.53	12.00 ^c,d^ ± 0.00	12.67 ^e^ ± 1.15	nd	18.67 ^c^ ± 2.89
Quercetin	38.47 ^e^ ± 1.90	nd	12.33 ^c,d^ ± 0.58	23.00 ^a^ ± 1.00	27.33 ^b^ ± 2.31	22.33 ^b^ ± 2.52
Caffeic acid	28.55 ^f^ ± 1.04	13.00 ^b,c^ ± 1.41	15.00 ^a^ ± 1.00	12.00 ^e^ ± 0.00	nd	nd
Antibiotic/antimicotic	na	17.00 ^a^ ± 1.00	14.00 ^a^ ± 2.00	19.50 ^b^ ± 0.50	34.00 ^a^ ± 2.00	19.67 ^b,c^ ± 0.94

VM—*Valeriana montana*; SR—*Salix retusa*; CH—*Campanula hercegovina*; F—flowers; L—leaves; B—barks; OB—old branches; YB—young branches; S—stems; nd—not detected; na—not analyzed. The data represent the means of three replicates (±standard deviation). The values within one column followed by the same letter do not differ significantly after the ANOVA post hoc Newman–Keuls analysis at a significance level of *p* < 0.01.

## Data Availability

Not applicable.
